# Supplementary material for: Self-care practice and associated factors among hypertensive patients who have follow-ups in public hospitals of Bahir-Dar City, Northwest Ethiopia, a mixed study
Source: PLoS One. 2025 May 9;20(5):e0317869. doi: 10.1371/journal.pone.0317869 (PMC12063846; doi:10.1371/journal.pone.0317869)
Supplement: Attachment — Submitted filename: Comment for author1.docx [file pone.0317869.s002.docx]

Title: Self-Care Practice and Associated Factors among Hypertensive Patients Who Have

Follow-Ups In Public Hospitals Of Bahir-Dar City, Northwest Ethiopia, A Mixed Study

Manuscript Number: PONE-D-24-13630

**Comment for authors**

General comment

Overall the research is good but has some sort of grammatical errors specially at the abstract and discussion section

Specific comments

Method section

Eligibility criteria

It is not correct please clearly specify your inclusion criteria

Sampling method

The sampling method for qualitative part is purposive but the samples were selected by considering some factors but it seems qota sampling

Please rethink over it because reader understand that it is qota sampling

Data collection

Have you checked the reliability of your data collection tool? If so how you checked?

Data analysis

The statistical significance of associations between variables was determined using odds ratios with a 95% confidence interval (CI) and p-values below 0.05

This statement is repeated please delete one of the two

Result

Instead of using tables as reference at each of statements please write in one paragraph and then use the table as reference and put it once

Discussion

The qualitative part of your finding is not discussed well please raise key issues and discuss with literatures

Declaration

Ethical consideration

Informed voluntary written and signed consent was obtained from all study participants prior to start data collection

why you choose written consent?
