# Supplementary material for: Self-care practice and associated factors among hypertensive patients who have follow-ups in public hospitals of Bahir-Dar City, Northwest Ethiopia, a mixed study
Source: PLoS One. 2025 May 9;20(5):e0317869. doi: 10.1371/journal.pone.0317869 (PMC12063846; doi:10.1371/journal.pone.0317869)
Supplement: Attachment — Submitted filename: point by point response Selfcare practice1111.docx [file pone.0317869.s003.docx]

# Author response to reviewers

**Submission** ID PONE-D-24-13630

**MANUSCRIPT Title**: **Self-Care Practice And Associated Factors Among Hypertensive Patients Who Have Follow-Ups In Public Hospitals Of Bahir-Dar City, Northwest Ethiopia, A Mixed Study**

| Reviewer 1 COMMENT | AUTHOR RESPONSE |
| --- | --- |
| **1.** Comment for authors **General comment** Overall the research is good but has some sort of grammatical errors specially at the abstract and discussion section | Thanks for your constructive comments: we have revised based on your comments |
| 2. **Specific comments** Method section Eligibility criteria It is not correct please clearly specify your inclusion criteria | Thanks for your constructive comments: we have revised based on your comments.   - All hypertensive patients aged 18 and older who had follow-ups at public hospitals in Bahir Dar city were included in this study. However, patients who were critically ill or had cognitive impairments during the data collection period were excluded. Additionally, patients with follow-up periods of less than six months were excluded, as a certain activity or behavior is assumed to be established only if it is practiced repeatedly for at least six months. |
| 3. Sampling method The sampling method for qualitative part is purposive but the samples were selected by considering some factors but it seems qota sampling Please rethink over it because reader understand that it is qota sampling | Thanks for your constructive comments: we have revised based on your comments.  Participants in the qualitative study was selected purposively on a quota basis. |
| **4.** Data collection Have you checked the reliability of your data collection tool? If so how you checked? | Thank you for your insightful comment. - We have checked reliability test for our tool, to assess reliability of the items individual Cronbach's Alpha was computed for individual latent variables and overall items of the tool. The overall Cronbach's Alpha consisting of 70 items was 0.902. The Cronbach's Alpha for the latent variables is also presented in table 6 as follows: S.No \| Variables \| Number of items \| Chronbach’s alpha; 1. \| Hypertension self-care practice \| 20 \| 0.879; 2. \| Knowledge \| 12 \| 0.789; 3. \| Social support \| 3 \| 0.766; 4. \| Self-efficacy \| 5 \| 0.945; 5. \| Perceived health status \| 12 \| 0.912; 6. \| Overall \| 52 \| 0.946 |
| *5.* Data analysis The statistical significance of associations between variables was determined using odds ratios with a 95% confidence interval (CI) and p-values below 0.05 This statement is repeated please delete one of the two | Thanks for your constructive comments: we have revised based on your comments |
| *6.* Result Instead of using tables as reference at each of statements please write in one paragraph and then use the table as reference and put it once | Thanks for your constructive comments: we have revised based on your comments. |
| **7.** Discussion The qualitative part of your finding is not discussed well please raise key issues and discuss with literatures | Thanks for your constructive comments: we have revised based on your comments. |
| ***8.*** Declaration Ethical consideration Informed voluntary written and signed consent was obtained from all study participants prior to start data collection why you choose written consent? | Thank you for your insightful comment.   - The preference for informed voluntary written and signed consent from study participants over oral consent is based on several key reasons:   1. **Documentation:** Written consent provides a clear record of the participant's agreement, serving as legal and ethical evidence that they were informed and willingly participated.  2. **Clarity and Understanding:** It allows researchers to communicate study details clearly, ensuring participants fully understand what they are agreeing to, which is essential for informed consent.  3. **Legal Protection:** Written consent includes language that safeguards participants' rights, including their ability to withdraw, confidentiality, and potential compensation.  4. **Accountability:** Requiring written consent encourages participants to engage actively and make conscious decisions about their involvement, enhancing the quality of research outcomes.  5. **Ethical Considerations:** Obtaining written consent aligns with ethical research principles by ensuring participants are fully informed and voluntarily consenting, thus maintaining research integrity.  While oral consent may be appropriate in emergencies, written consent is generally preferred to adequately inform participants and protect their rights. |
| Reviewer 2 COMMENT | AUTHOR RESPONSE |
| *1.* This manuscript is well-written, it could make a valuable contribution to literature on self-care practices of hypertension.  In result, it would be better if minimum and maximum age is also included along with average age of the participants  Thank you once again for your contributions. | Thanks for your constructive comments: we have revised based on your comments.   - The participants in the study ranged in age from a minimum of 25 years to a maximum of 89 years, with a mean age of 53.52 ± 13.7 years (standard deviation). |
